# Analysis of the effect of vibrational stress relief process parameters on 2024Aluminium alloy

**DOI:** 10.1016/j.heliyon.2024.e32052

**Published:** 2024-05-31

**Authors:** Hamidreza Mohammadhoseini Servak, Mehdi Jafari Vardanjani, Shahrouz Yousefzadeh

**Affiliations:** a Department of Mechanical Engineering, Aligudarz branch, Islamic Azad University, Aligudarz, Iran; b Department of Mechanical Engineering, Technical and Vocational University (TVU), Tehran, Iran

**Keywords:** Vibrational stress relief (VSR), Aluminium alloy, Process parameters, Experimental, Material

## Abstract

In principle, after all manufacturing processes are performed, a set of residual stresses occur in the product that have their particular distribution given the manufacturing process performed. The residual stresses must be removed to achieve the desired dimensional accuracy and quality. Among stress-relieving processes performed for a *piece* following the manufacturing process, we can refer to thermal and vibratory stress relief (VSR). Both methods perform the same function as they enter *a part or all of a piece* into the plastic phase, causing a fracture of residual stresses to be released with local plastic deformations. The process is as follows: The stress induced by thermal or vibratory loads is added to the residual stresses and exceeds the yield stress. This research, which is focused on VSR, aims to evaluate the effect of the main parameters of the VSR method, including load amplitude or amount, load application frequency, and cycle numbers. The general trend of the problem is that the VSR process is performed for a piece with residual stress, and the effect of the abovementioned parameters on reducing its residual stresses is evaluated.

## Highlights

• After performing all manufacturing processes, a set of residual stresses is created in the product.
• Residual stresses must be removed to achieve the desired dimensional accuracy and quality.
• Among the stress relief processes performed for a part following the manufacturing process is thermal and vibration stress relief (VSR).
• The stress caused by thermal or vibration loads is added to the residual stress and exceeds the yield stress.
• The main parameters of the VSR method include the amplitude or amount of load, the frequency of load application and the number of cycles.

## 1 Introduction

For many years, vibrational stress relief (VSR) has been used as an alternative to thermal stress relief (TSR) to reduce residual stresses. The current research includes a wide range of experiments, including vibration tests of pieces manufactured by processes such as casting, welding, and forging. Commercial processes usually include fastening the workpiece to an electric crank motor and vibrating it at different frequencies for a specific period. Research studies show that less distortion occurs after VSR is performed on metal pieces [1].

VSR is commonly used in machine tool beds and columns, tools used in the automotive and aerospace industries, pump chambers, water turbine equipment, paper-making machinery, equipment used in mines, large welded equipment, submarine components, and large and sensitive components. In compression to traditional furnace processes, in addition to having higher efficiency when performed on large sensitive parts, VSR has also developed both the range of manufacturable metal products and applications enjoying effective stress relief not only in terms of cost but also in terms of the dimensions or configuration of the pieces [[2], [3]].

The first study by McGoldrick et al. [4] was conducted on welded and cast frames. Although their study yielded no *useable quantitative results*, they concluded that VSR depends on the occurrence of plastic deformation phenomenon during the stress relief process. Therefore, *resonance amplitudes must be used to attain these goals. These results were confirmed by Moor using one-end-fixed conical beams*. He suggested that stress relief may depend on the degree of plastic deformation.

Buhler et al. [5] studied the effect of vibrations on cast and welded pieces. Although the results of their study were not of high certainty, they could decrease residual stresses up to 20 % more than before when the repeated stresses increase near the fatigue limit of the steel. They had to refrain from applying high-stress values to significantly eliminate residual stresses due to the limitation caused by the fatigue limit of the part.

Adoyan et al. [6] showed that cast iron retains its dimensions for a longer time as a result of the vibratory process.

To solve the problem of premature failure of the part, they proposed a combined solution, including a thermal vibration operation that was valuable at that time. Lokshin [7] could remove residual stresses up to 70 % in a preloaded cast aluminium ring. For this purpose, he used repeated stresses with resonance conditions and concluded that local relaxation of stresses during a vibration process disturbs the balance of the internal stresses, leading to stress redistribution and reduction of stress levels.

Perhaps until 1980, the best available research on this topic was conducted by Wonzney et al. [8]. They could reduce the residual stress in a shot-peened belt exposed to repetitive bending between two fixed supports by up to 33 %. Their work *was valuable in the way that* it could approximately provide an acceptable explanation for material softening behaviour. Moreover, the use of the obtained stress-strain curve for their given material showed that the reduction rate of residual stress can be predicted by knowing the value of initial stress, alternating stress amplitude, and final strain conditions.

Using the vibration process, Sagalevich et al. [9] could reduce the residual stresses occurring in the body of wagons made of mild steel sheets. They found a quasi-experimental relationship based on the equations governing the irreversible atomic rearrangement inside the crystal network under load.

Weiss et al. [10] tested a low-carbon disc with a diameter of 306 mm, which was welded to a 103 mm diameter disc through a sub-powder welding method. They observed a significant reduction in welding residual stresses. Samples were vibrated by a large laboratory vibrating device.

Claxton et al. [11] described the basic features of the VSR process and compared them with those of the thermal stress relief methods. The authors investigated the practical advantages and limitations of VSR using new examples compared with the TSR. They noted that there is little information about the VSR mechanism and how VSR affects dimensional stability and stress reduction. Therefore, it is the lack of knowledge that has led to a reduction in certainty regarding this process.

Botros [12] studied the effect of vibrations on the pattern of residual stress caused by welding mild steel structures. The results of his study showed that employing a proper vibrational process for a welded mild steel structure significantly reduced the residual stress peak values in the welded structure for 15 min. The results also showed that the residual stresses were the result of the nonuniformity of plastic deformations and that vibrations reduce the degree of nonuniformity by applying dynamic stress.

In his Ph.D. thesis, Dawson [13] invented a technique for the uniform distribution of residual stress in one-end-fixed beams. The obtained results showed that the VSR methods are capable of nearly complete removal of surface stresses in the two types of studied steel and *aluminum alloy hot and cold-rolled* samples. The results obtained for these cases were similar to the extent to which the authors came to the belief that the results of most metal structures undergoing VSR will be the same. The results also showed that the maximum stress relief (20–50 %) occurs in the first cycle, a lower level of stress relief (25–30 %) occurs in the second cycle, and the remaining stress is relieved in the subsequent cycles. In addition, Munsi et al. [14] conducted extensive research on residual stress relief for welded parts by applying high-frequency vibration and could attain extensive stress relief.

Kuang [15] investigated the VSR using the finite element model with ANSYS software. This research was conducted by assuming the expansion of stresses and neglecting the expansion of welding stresses. Moreover, Aoki [16] applied different frequencies of vibrational load on the weld line between two welded plates and measured welding residual stress by using the X-ray diffraction technique and found that the residual stresses were reduced. Finally, he evaluated his work using an analytical method. The employed analytical model consists of mass and preloaded springs with elastoplastic characteristics. A reduction in residual stress was also observed in this model.

Vergeer [17] studied the characteristics of flow-induced vibrations in plate heat exchangers. He found that although the effect of dimples on channel walls has been well evaluated, the elastic vibration of the body has not been sufficiently studied. Therefore, a simple aerodynamic model was developed to determine the combined characteristics of the body (plate) and the flow of the plate-induced vibration.

Kahya et al. [18] proposed a finite element model based on the first-order shear deformation theory for the analysis of free vibration and buckling of functionally graded (FG) sandwich beams. They considered two types of sandwich beams: a) a Type A sandwich beam with FG faces and a homogeneous ceramic core and b) a Type B sandwich beam with homogeneous metal and ceramic faces and an FG core. The abovementioned sandwich beam types were studied in this research. The effect of vibration on each type was evaluated and revealed significant results. A similar study by Kilic et al. [19] specifically investigated the simple type of FG beams. Although these studies are focused on FG beams, their output is only related to sandwich beams, limiting their applications.

In [20], the authors investigated the effect of thermoforming parameters on the structure and mechanical properties of the helical gearbox cover made of 356A alloy. In this research, the effect of forming parameters such as solid fraction, temperature, pressure, punching speed, and heat treatment on the microstructure and mechanical properties of the sample was investigated. The results show that by changing the pressure from 100 to 150 MPa, the average diameter of the particles decreases by about 7 % and the shape coefficient also increases. Also, with increasing temperature, hardness and press force decreased by 13 and 21 %, respectively. The results show that with increasing temperature and storage time, the diameter of particles and initial phase increases, and hardness decreases. If the holding time increases from 5 min to 30 min, the particle diameter increases by 74 %.

Jafari Vardanjani et al. [21] performed the residual stress reduction process practically and theoretically for 2024 aluminium alloy using vibratory methods and analysed the effective parameters. They concluded that the sub-resonant vibrations with proper duration will significantly affect the stress relief of this alloy. The theoretical and computational part of this research was developed in the next study by Jafari et al. [21], and new mathematical relations based on frequency, amplitude, and time parameters were obtained. These relations can be widely used for some alloys depending on the experimental or working conditions.

Recently, research studies on thermal-vibratory stress relief (TVSR) and its comparison with other processes have been conducted independently. According to the study by Gao et al. [22], this combined process has made the residual stresses in aluminium alloy more uniform and has outperformed the thermal stress relief process by 81 %. As the authors stated, this combined process also has a slight effect on hardening and metallographic structure.

In another study by Gao et al. [23], 7075 aluminium alloy samples underwent TVSR, and a finite element analysis was conducted on this alloy. The obtained results showed that TVSR outperformed each of the thermal and vibratory processes alone. The results also showed that TVSR does not affect the grain size, but it affects dislocations.

Gao et al. [24] also conducted a study to compare the effect of TVSR and each of the TSR and VSR processes on Ti6Al4V samples. The results of this study also showed no significant effect on the grain shape and phases. Finally, it was concluded that this combined process can reduce the stress of the Ti6Al4V sample at a lower cost and in less time.

Although TVSR can be considered a proper method to improve the quality of some aluminium alloys given the abovementioned studies, its disadvantages should not be overlooked. The first problem related to this method is the needed equipment and the relatively high cost of performing this process [25]. The equipment should act in such a way that not only meets the need for planning thermal and vibratory parameters versus time but also provides sufficient space for parts. Furthermore, this mechanism has not yet been deeply analysed numerically and experimentally to discuss all its aspects, and further research is needed. Moreover, some studies have highlighted the low effect of this process. For example, in their study, Song et al. [26] concluded that TVSR has been incapable of relieving residual stresses in large AA2219 rings.

## 2 Research method

Although according to previous research [4,12,27], one of the most common methods for applying vibration in the VSR method is the use of crank motors and accelerometer sensors to apply and measure the process, other innovative methods [28,14,20,22] can be employed for this process. What is important in these methods is that the workpiece should be fixed in such a way that it is isolated from the surrounding environment and tools as much as possible while having the needed degree of freedom for vibration. In cases in which vibration isolation of the workpiece is not possible, the vibrating unit should be as close as possible to the work area. To transfer vibrational energy, the vibrating unit should be directly engaged with the workpiece, which can be done with the help of restraints. After these conditions are met, the piece should be vibrated with appropriate parameters of amplitude, time, and frequency. Once these conditions are met, the piece should be vibrated with appropriate parameters: amplitude, time, and frequency.

Considering the advantages and limitations of the VSR and the current limitations of the TSR, the TSR's capability for reducing residual stresses and improving the dimensional stability of the parts has been proven to a large extent. Moreover, the VSR method can be combined with manufacturing processes as a lateral method. Performing TSR on some materials is not, however, recommended according to previous research [15,22,23], indicating the *inherent limitation of the* TSR. Additionally, it is likely that some steel alloys undergo unfavourable microstructural changes and *experience loss of mechanical properties* and cracking after TSR. Distortions, oxidation, loss of corrosion resistance, and intergranular cracks are also among the potential risks posed by performing TSR on some alloys [22]. However, more comprehensive studies, comparisons, and analyses are needed to be conducted for the complete replacement of TSR, which will need access to different equipment and alloys.

Considering the abovementioned studies, it can be found that despite its practical and operational advantages in the manufacturing process, VSR has not been deeply and parametrically analysed, or scant research has been conducted in this regard. Therefore, the main aim of the present study is to analyze the effect of the main parameters of the VSR on the reduction rate of the workpiece's residual stress induced by the manufacturing process. This study was conducted in three parts: simulation, experimental, and theoretical analyses. For simulation, the parameters of the VSR process were completely evaluated (48 experiments) with Abaqus software for a one-end-fixed beam made of the given material for all three parameters (cycle, frequency, and load), including two four-level factors and one three-level factor, and the optimal levels of parameters were obtained [[29], [30]]. The results of this simulation and experimental test conducted on the one-end-fixed beam under oscillating loading were analysed. This comparison uses the hardness properties and elasticity coefficient of the object obtained by the *nano indenter device.* In the analytical part, first a *pure bending moment* $M_{1}$, which introduces the beam into the plastic phase up to the depth $h_{1}$, was applied to the beam. After unloading, the residual stress distribution was obtained using elastic relations, ideal strain‒stress curves, and geometrical similarities in the beam [31].

The residual stress distribution in the beam was obtained with the help of elastic relations, ideal stress-strain diagrams, and geometric similarities. Next, the reverse bending load ($M_{2}$) that introduces the beam into the plastic phase up to the depth $h_{2}$ was applied to the beam. The residual stress distribution was obtained after releasing the beam. Finally, the obtained results were analysed and compared.

### 2.1 Simulation, experimental, and theoretical analysis tests

#### 2.1.1 Finite element analysis (FEA)

**Fig. 1:**
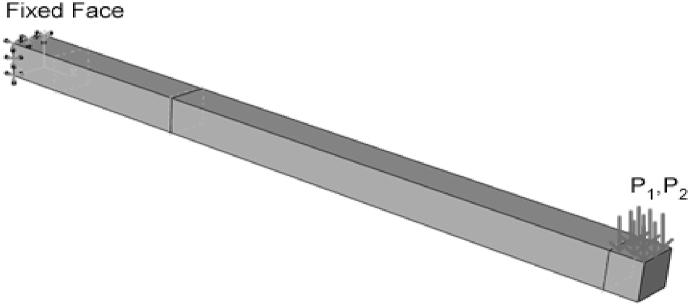
Beam simulated model, boundary conditions, and how loads are applied.

For the simulation of the FEA problem, a one-end-fixed beam with dimensions of 10 × 10 × 20 mm was modeled in Abaqus software. In the first stage, the residual stress distribution was created in the beam by applying the *moment* $M_{1}$. This was done by imposing the pressure $P_{1}$ on part of the beam free area and subsequently the bending moment $M_{1}$ (Fig. 1).

**Fig. 2:**
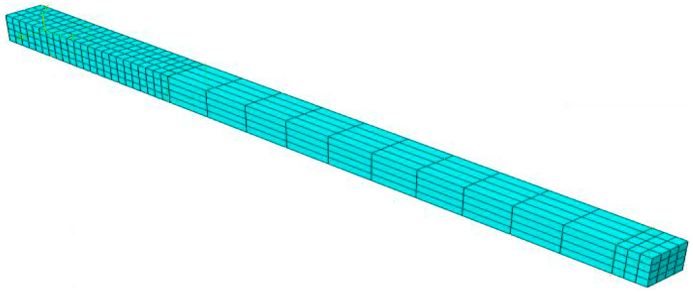
Displaying elements in the FEA environment (simulation).

In the next step, the moment $M_{1}$ ($P_{1}$) was removed, and the moment $M_{2}$ was applied in a completely reversible way [32]. The parameters, including time, amplitude (load value),and frequency, were considered in $M_{2}$ ($P_{2}$) using tabulated data. The assumed elements in the given beam are shown in Fig. 2.

#### 2.1.2 Experimental test

**Fig. 3:**
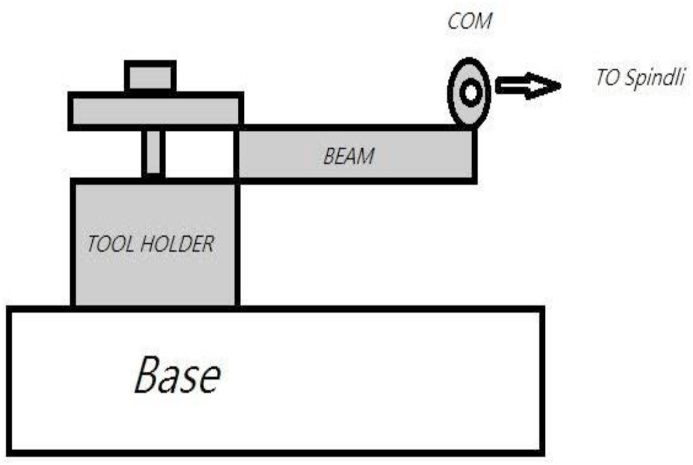
Configuration used to impose oscillating load on the beam.

**Fig. 4:**
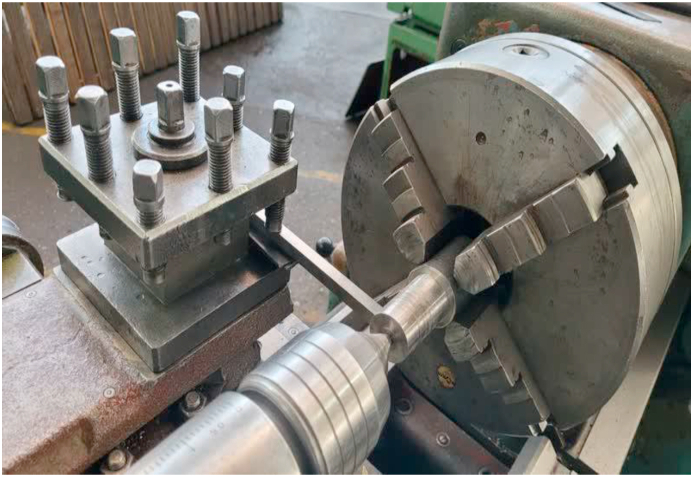
Imposing oscillating load on the beam in the experimental test.

First, a beam with dimensions of 10 × 10 × 230 mm was prepared as the workpiece. The beam was cut using a band saw, and the residual stresses induced by this process were overlooked. Two other beams already rolled under the same conditions in the factory were prepared with rolled dimensions of 10 × 10 × 230 mm. The VSR process was performed on one of the beams. Fig. 3 displays the configuration used to conduct this experiment, and Fig. 4 shows a picture of the process [33].

The manual lathe machine was used to impose the oscillating load. The engaged part of the beam was clamped using the tool holder of the lathe machine. The other end of the beam was placed in front of the spindle. The *camshaft* with the desired crank degree was put in the chunk lathe to apply the given torque. In this way, the oscillating load was generated by rotating the shaft and the cam-shaped member-beam contact [34]. Therefore, the amplitude equals the range of the cam radial movement. The frequency of the load imposition was determined by adjusting the rotational speed of the spindle. Finally, three beams with specific conditions were obtained. Table 1 shows the beam's specifications (see Table 2).

**Table 1:** Performed process sequence and beam specification.

No.	The process performed on the beam	Dimensions
1	–	10×10×230mm
2	Rolling	10×10×230mm
3	Rolling and vibratory stress relief	10×10×230mm

**Table 2:** shows the position specification and type of loading for one of the tested points by the nanoindenter device.

Indentation # 1	
**Indentation parameters**	
Instrument	NHTX S/N: 01-03119
**Hardware settings**	
Approach speed	1500 Nm/min
Delta Slope Contact	100 %
X Position	17.96 mm
Y Position:	11.49 mm
**Measurement**	
Acquisition Rate	10.0 [Hz]
**Linear Loading**	
Max load	50.00 Nm
Loading rate	100.00 Nm/min
Unloading rate	100.00 Nm/min
**Indenters**	
Type	Berkovich
Material	Diamond

**Fig. 5:**
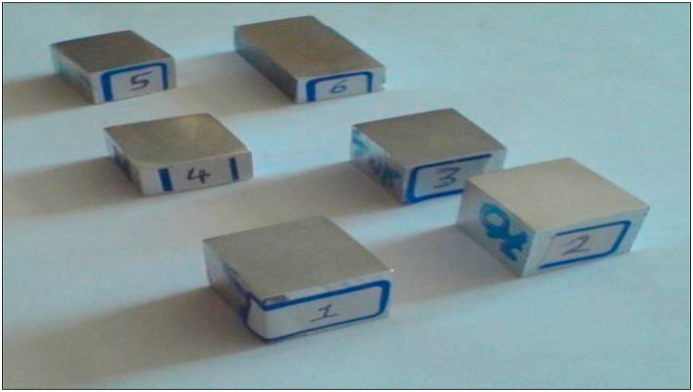
Samples under test.

After the test was conducted, a sample with dimensions of 10×10×10mm was isolated from the middle part of the beam (Fig. 5). The upper and lower surfaces of these three specimens were polished to conduct the nano indenter test and investigated using the nano indenter device. This device gives values for hardness properties, modulus of elasticity, and elastic and plastic work done during indentation. The approximate amount of residual stress can be obtained by measuring the abovementioned properties and converting them to stress values at points under indentation. Moreover, it should be noted that according to Ref. [27] and the experiments carried out in the studies by Kahya et al. [18], Yang [35], and Jafari et al. [20] and experiments conducted by the Stress Relief Engineering (SRE) Co. [36], measuring the sample hardness can be considered a standard method to evaluate the effect of stress relief processes on reducing the residual stress rate provided that the number of experiments is sufficient and the given alloy is in a particular group of aluminium and steel alloys.

#### 2.1.3 Theoretical analysis

**Fig. 6:**
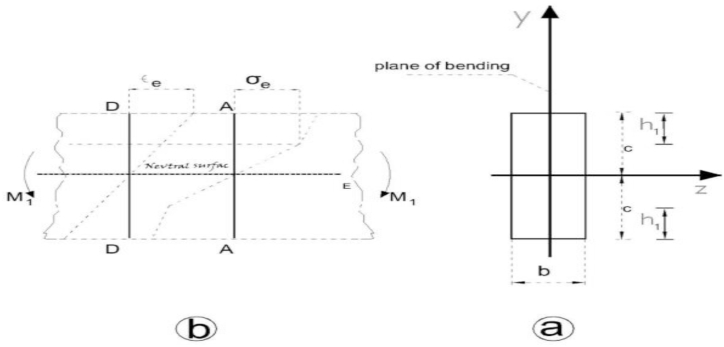
Stress distribution when the load is applied and the beam cross-section [37] (a) The beam cross-section that shows (b) The distribution of stress and strain on the beam cross-section when the initial moment is applied.

The residual stresses can be defined as stresses created in an object without the presence of external forces. In this case, the stress system should be in static balance. The total force on each element of this object should be zero. In line with obtaining the relations related to the members with residual stress, any attempt made to gather different mechanical properties of elastoplastic solids in a mathematical model to be considered as the basis for solving the theoretical problems under discussion will be complicated. Therefore, simpler models that only propose the necessary properties of the problem should be used. Moreover, the need to simplify math often leads to idealization in mathematical relations about the mechanical properties of the model. In some cases, it is possible to solve the problem of plasticity based on stress-strain diagrams, as shown in Fig. 6. In this problem, we assume that the initial residual stresses have been created by using pure bending. The residual stresses are then removed by alternating quasistatic and dynamic loading.

#### 2.1.4 Residual stress generation

**Fig. 7:**
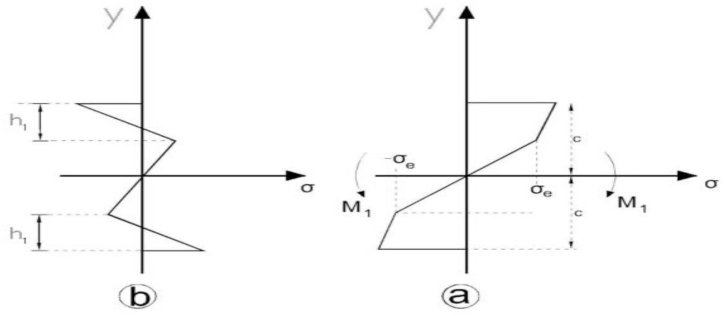
Stress distribution while the moment is applied and after that [20] (a) stress distribution while the bending moment is applied (b) Distribution of the initial residual stress when the bending moment is removed.

**Fig. 8:**
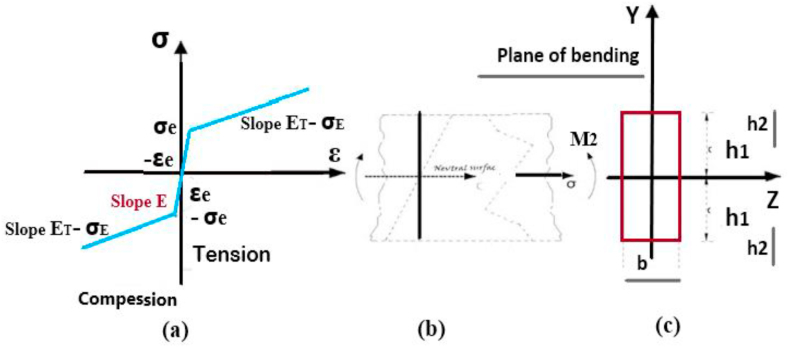
Stress distribution while the load $M_{2}$ is applied and the beam cross-section [20] (a) Beam stress-strain curve [37] (b) Distribution of the stress and strain in the beam cross-section when the bending moment is reversed (c) Beam cross-section that shows $h_{1}$ and $h_{2}$.

First, the straight beam without the initial residual stresses, whose *tensile and compressive stress-strain diagram* is shown in Fig. 7(c), was considered. This diagram shows that the material has an elastic or endurance limit of $\sigma_{e}$ related to the strain of the elastic limit of $\varepsilon_{e}$, and its *compressive stress-strain diagram is similar to the stress-strain diagram obtained for tension.* According to 6 (a), the given beam with a square cross-section and a width of b and depth of 2c was under bending moment $M_{1}$ at both ends around the Oz axis parallel to the b face. In this case, *the neutral axis* divides the area of the *cross-section* into two *halves* due to the double symmetry that exists. Fig. 8 (B) shows the strain changes expressed by relation (3). The maximum stress occurs in external fibers which will be tensile at y=c and compressive at y=−c. When the maximum stress equals the yield stress ${\;\sigma }_{e}$, the bending moment $M_{e}$ is the maximum moment that the *cross-section* can *endure* before yielding. $M_{e}$ is the bending moment of yielding or elastic moment. The $M_{e\;}$ value can be obtained using the bending theory as follows:

(1) $$M_{e}=\frac{\sigma_{e}I}{c}$$

where I is the inertia moment of the beam around axis z, and its value equals [38]:

(2) $$I=\frac{2}{3}bc^{3}$$

By substituting I from relation (2) into relation (2), we have [34]:

(3) $$M_{e}=\frac{2}{3}\sigma_{e}bc^{2}$$

Here, it is assumed that the stress at the outermost points exceeds the yielding stress ${\;\sigma }_{e}$, creating the strain of the elastic limit $\varepsilon_{e}$.

Releasing the bending moment that causes nonelastic stresses to be generated creates residual stresses at the cross-sections where nonelastic strains occur. The residual stress value can be obtained using the following principle. When a load, that has generated nonelastic strains, is relieved, the part acts elastically while the load is released. Therefore, the stresses and strains related to the unloading cycle can be obtained using the typical elastic stress relations. Here, it is assumed that the stresses imposed on each layer will not exceed two times the yielding stress in any part of the beam. As the value of the stresses generated in the loading phase is added algebraically to the residual stresses, the value of the residual stresses can be obtained.

Considering the obtained results and analyses, the distribution of the initial residual stress $\sigma_{ir}$ in the beam is obtained as follows:

(4) $$\sigma_{\text{ir}}=\left(\frac{y}{c-h_{1}}\right)\sigma_{e}-\frac{M_{1}y}{I},0\leq y\leq c-h_{1}$$

(5) $$\sigma_{\text{ir}}=\left(1-\beta +\frac{\beta y}{c-h_{1}}\right)\sigma_{e}-\frac{M_{1}y}{I},c-h_{1}\leq y\leq c$$

Figure (8) (B) shows the distribution of the initial residual stress along the beam depth, which is expressed by relations (7) and (8). The residual stresses present in the external fibres are in the same direction as the stresses generated by reverse loads.

#### 2.1.5 Reduction of residual stresses

If the load is applied in the reverse direction after releasing the load that has induced nonelastic strains, the nonelastic strains are found to be generated by a smaller load in the opposite direction. The justification for the small amount of this reverse load, which has generated nonelastic strains, is that according to Fig. 8 (a) and 8 (b) after the initial load is removed, the residual stresses occurring in the outer layers of the beam are in the opposite direction of the load that has induced them. Therefore, the initial residual stresses in the outer layers are in the same direction as the stresses induced by the reverse load. In this section, we address the stress distribution along a beam of square cross-section when the load is reversed. The reverse load is smaller than the initial load applied to generate the initial residual stresses. A beam that is under the bending moment $M_{2}$ from the endpoints around the axis Oz, in parallel with face b, is considered. This bending moment $M_{2}$ is in the opposite direction of the moment that induced the initial residual stresses (Fig. 8 (a) and 8 (b)). The stress distribution created in the upper half of the beam is similar (and opposite) to the stress distribution in the lower half because the tensile stress-strain curve has been regarded as similar to the compressive curve. Therefore, we only need to be aware of the stress distribution in one half of the beam. Considering *the neutral axis, such as the initial bending moment that induces initial residual stresses, this neutral axis* divides the area of the *cross-section* into two *halves* because the cross-section is symmetrical around the neutral axis, and the stresses change as an odd function in the thickness of the beam. Fig. 8 (b) shows the linear changes in the strain expressed by relation (2). The maximum tensile and compressive stresses occur at y=−c and y=c, respectively. It should be noted that the yield induced by the reverse load at both sides penetrates up to the depth $h_{2}$ of the beam, which is less than the depth $h_{1}$ related to the initial loading to generate initial residual stresses because it is assumed that the value of the initial load is larger than that of the reverse load.

The residual stress distribution after releasing the reverse bending moment can be obtained via the principles used to calculate the initial residual stresses in the beam before applying the reverse load. This means that once a load that has induced nonelastic strains is released, the part acts elastically. The elastic stresses related to releasing the reverse load can be obtained using the following relation:

(6) $$\sigma_{u2}=\frac{M_{2}y}{I}$$

Where $\sigma_{u2}$ is the elastic stress related to unloading. By combining the relations obtained from calculations, the distribution of the final residual stress in the beam after unloading the reverse bending moment is obtained by the following relations [39]:

(7) $$\sigma_{fr}=\left(\frac{1}{c-h_{1}}-\frac{2}{c-h_{2}}\right)y\sigma_{e}+\frac{M_{2}y}{I},0\leq y\leq c-h_{1}$$

(8) $$\sigma_{fr}=\left(1-\beta +\frac{\beta y}{c-h_{1}}-\frac{2y}{c-h_{2}}\right)\sigma_{e}+\frac{M_{2}y}{I},c-h_{1}\leq y\leq c-h_{2}$$

(9) $$\sigma_{fr}=\left(-1+\beta +\frac{\beta y}{c-h_{1}}-\frac{2\beta y}{c-h_{2}}\right)\sigma_{e}+\frac{M_{2}y}{I}\;c-h_{2}\leq y\leq c$$

The residual stress distribution after releasing the reverse bending moment can be obtained via the principles used to calculate the initial residual stresses in the beam before applying the reverse load. This means that once a load that has induced nonelastic strains is released, the part acts elastically. Table 4 shows The average reduced stress value (pa) for each experiment (AL2024).

**Table 3:** Mechanical properties of aluminium.

Parameter	Value
Modulus of elasticity (E)	73.1
Tangential elastoplastic modulus ($E_{T})$	0.7
yield stress (elastic limit) ($\sigma_{e}$)	324
Density (ρ)	2780
The ratio of tangential elastic modulus to elastoplastic modulus (β)	0.0098

**Table 4:** The average reduced stress value (pa) for each experimental (AL2024).

		Time(s)			
Frequency(Hz)	Moment(Nm)	5	10	15	20
**49**	50	7,975,766	8,208,256	10,254,000	7,534,990
	60	11,002,222	13,124,000	11,913,044	11,148,254
	70	10,218,316	12,815,506	12,193,638	10,398,488
**80**	50	4,985,784	4,027,390	4,143,992	1,478,014
	60	8,513,518	6,598,940	4,571,988	2,498,880
	70	7,406,628	5,132,192	2,715,414	372,100
**95**	50	7,178,370	5,648,326	4,177,046	4,037,784
	60	9,410,440	6,606,008	4,305,188	2,036,826
	70	7,514,812	4,805,196	2,290,192	−385,620
**120**	50	4,888,486	3,713,090	2,786,546	1,426,888
	60	9,410,440	4,805,196	4,090,854	2,267,058
	70	7,514,812	5,528,406	3,138,508	605,956

## 3 Discussion and results investigation

### 3.1 Results of finite element analysis

Alternative loading has been applied with a frequency lower than the normal frequency and vibration without damping to a one-end-fixed beam. The fundamental natural frequency is obtained using the following relation:

(10) $$\omega_{n}=\frac{\pi^{2}}{L^{2}}\sqrt{\frac{EI}{\rho A}}$$

where L is the beam length, A is the area of the cross-section E is the elastic modulus (also known as the *modulus of elasticity*), ρ is the density, and I is the inertia moment of the beam cross-section around the axis z, and its value is obtained by the following relation:

(11) $$I=\frac{b^{4}}{12}$$

Therefore, for I, we have:

(12) $$I=1.0583\times 10^{-9}\;m^{4}$$

The normal frequency of the beam is thus equal to:

(13) $$\omega_{n}=\frac{\pi^{2}}{{\left(0.23\right)}^{2}}\sqrt{\frac{7.31\times 10^{10}\times 1.05833\times 10^{-9}}{2780\times 0.000161}}=\begin{array}{c} 2450 \end{array}\frac{rad}{\sec}=\begin{array}{c} 390Hz \end{array}$$

**Fig. 9:**
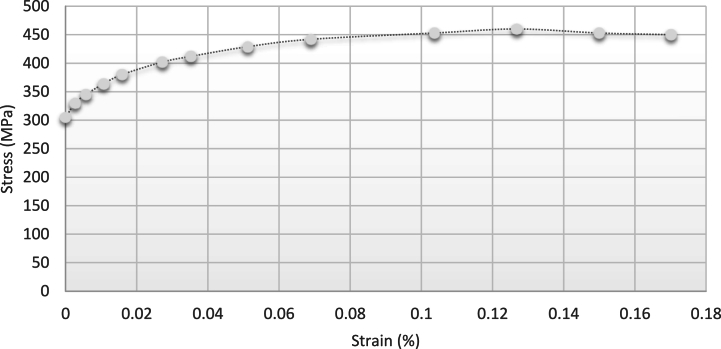
Stress-strain diagram of the 2024 aluminium plastic part.

Therefore, the needed frequency in the conducted test has been considered lower than this value to apply the *sub-resonance* alternative loading. The parameters of the tested material are shown in Table 3-1. Fig. 9 shows the stress-strain curve for the part that enters the plastic phase. The following values were used to analyze the vibrational load:

(14) c=5mm

(15) b=12.7mm

(16) $$M_{1}=100\;\text{Nm}$$

(17) $$M_{2}=50,60,70\;\text{Nm}$$

**Fig. 10:**
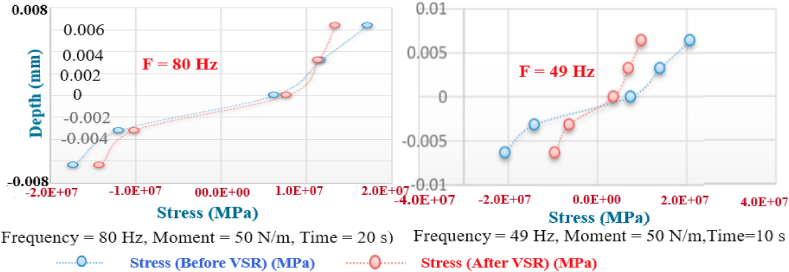
Diagram of the residual stress distribution on the cross-sectional area of the beam before and after applying an oscillating load in the simulation environment for 2024 aluminium (frequencies = 49.80 Hz, moment = 50 N/m, times = 10 s, 20 s).

**Fig. 11:**
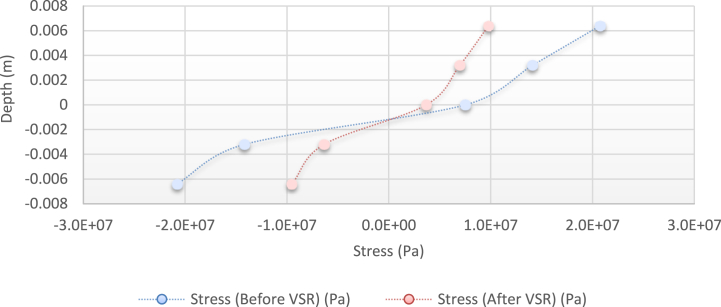
Diagram of the residual stress distribution on the cross-sectional area of the beam before and after applying an oscillating load in the simulation environment for 2024 aluminium (frequency = 80 Hz, moment = 50 N/m, time = 10 s).

**Fig. 12:**
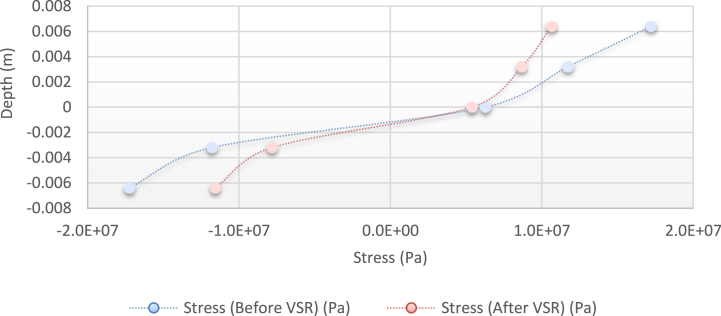
Diagram of the residual stress distribution on the cross-sectional area of the beam before and after applying an oscillating load in the simulation environment for 2024 aluminium (frequency = 95 Hz, moment = 50 N/m, time = 10 s).

**Fig. 13:**
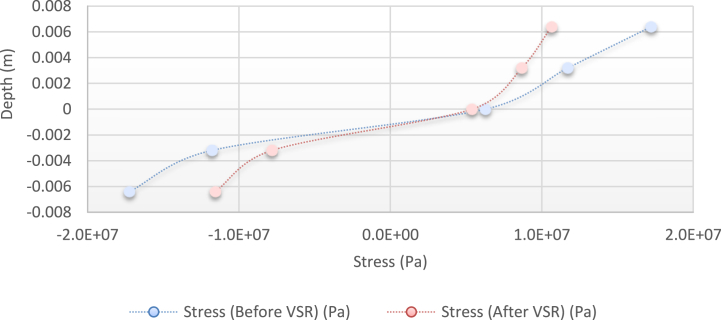
Diagram of the residual stress distribution on the cross-sectional area of the beam before and after applying an oscillating load in the simulation environment for 2024 aluminium (frequency = 120 Hz, moment = 50 N/m, time = 10 s).

Fig. 10, Fig. 11, Fig. 12, Fig. 13 show the distribution of the initial residual stress induced by the moment $M_{1}$ and the reduced residual stress after applying the alternative load in specific values on the finite element model of 2024 aluminum with specifications and distribution of the abovementioned initial residual stress for time 10 s and load 50 N/m for 4 different frequencies.

**Fig. 14:**
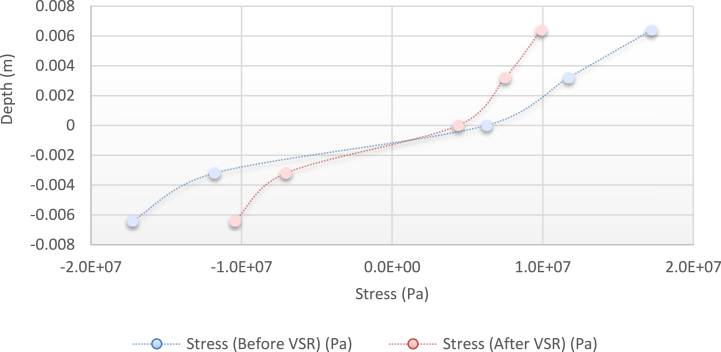
Diagram of the residual stress distribution on the cross-sectional area of the beam before and after applying an oscillating load in the simulation environment for 2024 aluminium (frequency = 80 Hz, moment = 50 N/m, time = 5 s).

As seen from the comparison of the 4 diagrams shown in Fig. 2, Fig. 3 to Fig. 3, Fig. 5, the residual stress reduction at a frequency of 49 Hz assigns a greater value to itself. This frequency thus yields a better result in the alternative stress relieving process, indicating that stress relief by low frequency for load = 50 N/m and time = 10 s yields better results. Although this frequency is very different from the calculated normal frequency, we aim to study sub-resonance VSR. Moreover, the effect of load and time should not be overlooked because these two parameters sufficiently affect the occurrence of this phenomenon. Now, we consider the element "time" and compare the results. The diagrams shown in Fig. 14, Fig. 16 display these comparison results (see Fig. 17).

**Fig. 15:**
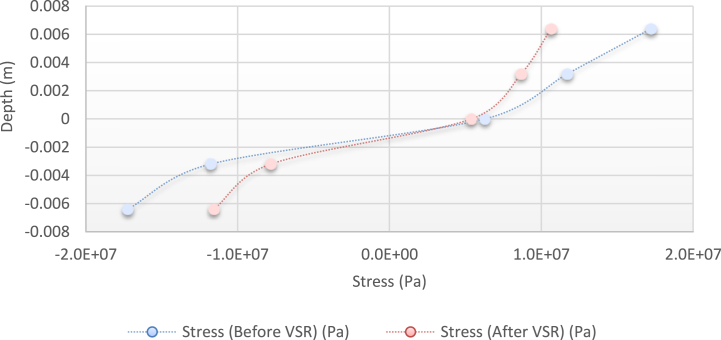
Diagram of the residual stress distribution on the cross-sectional area of the beam before and after applying an oscillating load in the simulation environment for 2024 aluminium (frequency = 80 Hz, moment = 50 N/m, time = 10 s).

**Fig. 16:**
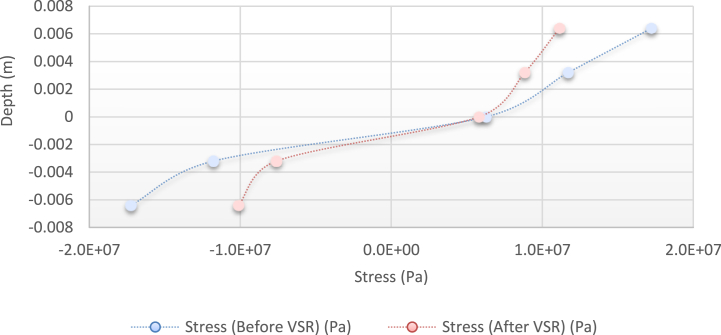
Diagram of the residual stress distribution on the cross-sectional area of the beam before and after applying an oscillating load in the simulation environment for 2024 aluminium (frequency = 80 Hz, moment = 50 N/m, time = 15 s).

**Fig. 17:**
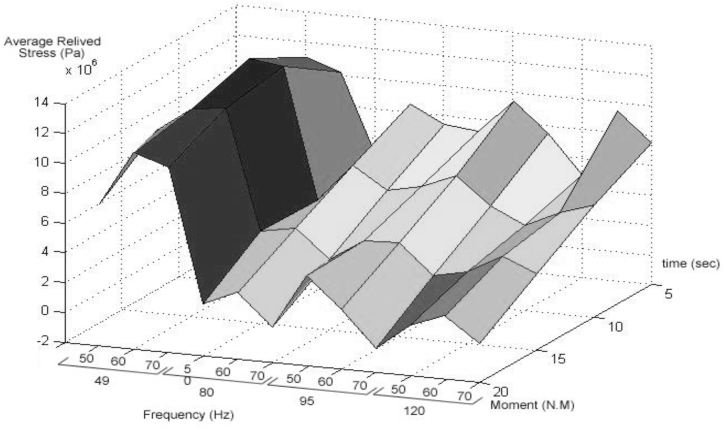
Mean residual stress reduction in the simulation environment for each experimental situation stress reduction in the simulation environment each experimental situation.

Residual stress distribution on the cross-sectional area of the beam before and after applying an oscillating load in the simulation environment for 2024 aluminium (frequency = 80 Hz, moment = 50 N/m, time = 10 s)

Fig. 14, Fig. 15, Fig. 16 show the residual stress distribution at a frequency of 80 Hz for a load of 50 N/m. As can be observed, time = 5 s shows a greater reduction compared with other times. The justification for this goes back to the principle of significant stress relief in the initial cycles. As mentioned above, a significant reduction in residual stress occurs in the first cycles of alternative loading. By looking at the above diagrams, we find that not only did a significant reduction occur during the first cycles (5 s) but also that the continuation of the stress relief in the subsequent cycles adversely affected the stress relief process.

**Fig. 18:**
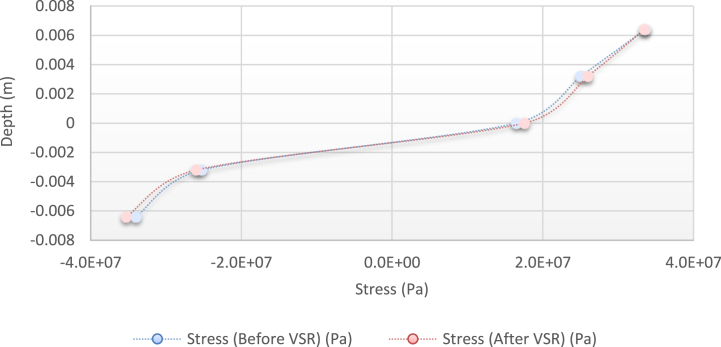
Diagram of the residual stress distribution on the cross-sectional area of the beam before and after applying an oscillating load in the simulation environment for 2024 aluminium (frequency = 80 Hz, moment = 90 N/m, time = 5 s).

**Fig. 19:**
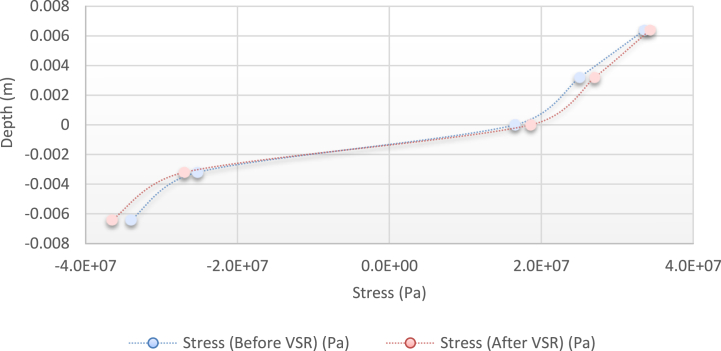
Diagram of the residual stress distribution on the cross-sectional area of the beam before and after applying an oscillating load in the simulation environment for 2024 aluminium (frequency = 80 Hz, moment = 90 N/m, time = 10 s).

If the mean values of the residual stress reduction in the cross-section for each experiment (related to each parameter) are as shown in Table 9, the optimum conditions to experiment can be obtained. As can be observed in Fig. 18, the obtained results show that the residual stress reduction increases by moving toward lower frequencies and less time, and more desirable results are obtained. Considering the load amount, it should be said that changes in the load amount have caused no significant effect on this reduction, but the middle level of the load factor has yielded better results (see Fig. 19).

**Table 5:** Mean values of experimental test, Frequency = 49 HZ, Amplitude = 0.8 mm, Time = 60s.

Specimen	Mean hardness (Vickers)
**Before rolling**	212.191
**Rolled**	230.786
**Rolled and Vibrated**	185.655

**Table 6:** Mean values of experimental test, Frequency = 80HZ, Amplitude = 0.8 mm, Time = 60 s.

**Specimen**	**Mean hardness (Vickers)**
**Before rolling**	212.191
**Rolled**	230.786
**Rolled and Vibrated**	196.725

**Table 7:** Mean values of experimental test, Frequency = 95HZ, Amplitude = 0.8 mm, Time = 60s.

**Specimen**	**Mean hardness (Vickers)**
**Before rolling**	212.191
**Rolled**	230.786
**Rolled and Vibrated**	199.635

**Table 8:** Mean values of experimental test, Frequency = 120HZ, Amplitude = 0.8 mm, Time = 60s.

**Specimen**	**Mean hardness (Vickers)**
**Before rolling**	212.191
**Rolled**	230.786
**Rolled and Vibrated**	206.360

**Table 9:** Mean values of experimental test, Frequency = 49HZ, Amplitude = 0.8 mm, Time = 80s.

**Specimen**	**Mean hardness (Vickers)**
**Before rolling**	212.191
**Rolled**	230.786
**Rolled and Vibrated**	189.652

### 3.2 Results of the experimental part

**Fig. 20:**
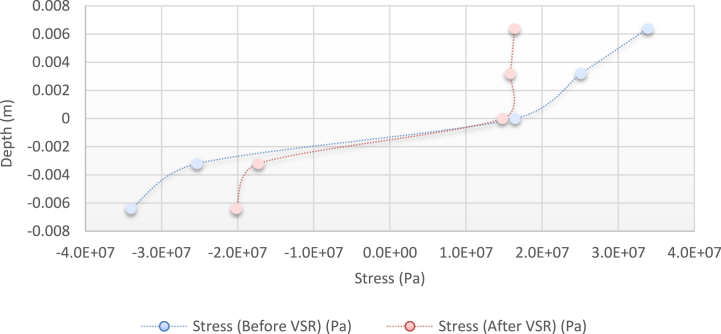
Diagram of the residual stress distribution on the cross-sectional area of the beam before and after applying an oscillating load in the simulation environment for 2024 aluminium (frequency = 80 Hz, moment = 90 N/m, time = 15 s).

**Fig. 21:**
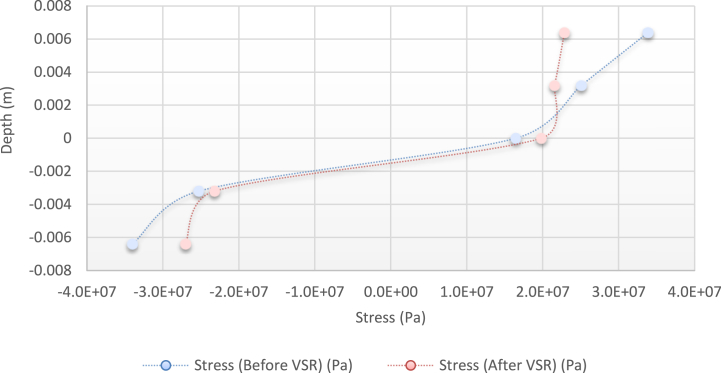
Diagram of the residual stress distribution on the cross-sectional area of the beam before and after applying an oscillating load in the simulation environment for 2024 aluminium (frequency = 80 Hz, moment = 90 N/m, time = 20 s).

The results of the experimental part for the given beam were tested for the beams with dimensions of 10 × 10 × 225 mm with a configuration similar to the finite element conditions and theoretical analysis. There are differences in the experiments conducted in Ref. [13] compared with the experiments carried out in this project.(see Fig. 20, Fig. 21)

• The rolling process was used instead of the milling process.
• Stresses were measured using another method.

An indenter test was performed on three samples made of this material. The obtained results are shown in Table 5, Table 6, Table 7, Table 8 for 4 different frequencies and in Table 9, Table 10, Table 11 for 4 different times.

**Table 10:** Mean values of experimental test, Frequency = 49HZ, Amplitude = 0.8 mm, Time = 100s.

**Specimen**	**Mean hardness (Vickers)**
**Before rolling**	212.191
**Rolled**	230.786
**Rolled and Vibrated**	186.803

**Table 11:** Mean values of experimental test, Frequency = 49HZ, Amplitude = 0.8 mm, Time = 120 s.

**Specimen**	**Mean hardness (Vickers)**
**Before rolling**	212.191
**Rolled**	230.786
**Rolled and Vibrated**	175.523

**Fig. 22:**
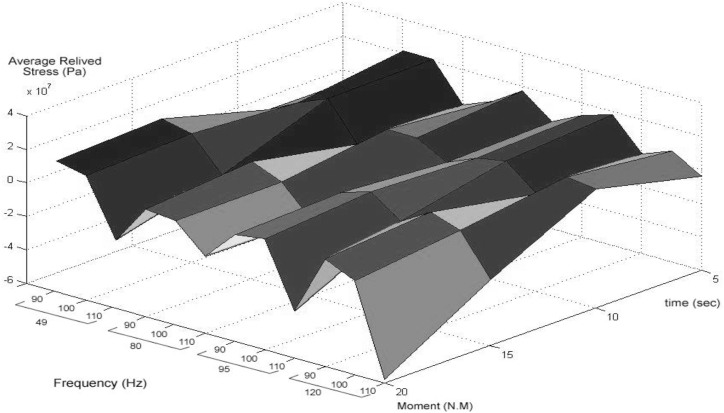
Mean residual stress reduction in the simulation environment for each experimental situation.

**Fig. 23:**
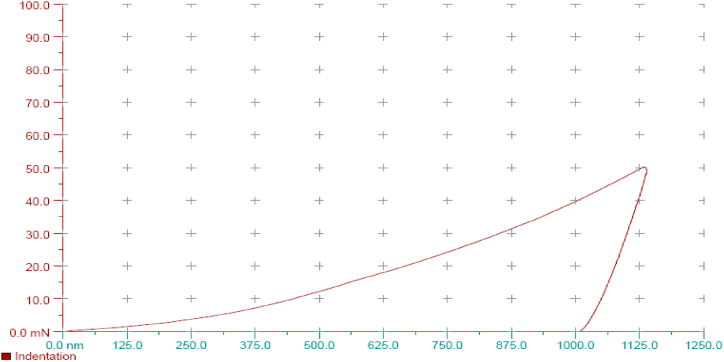
Diagram of the force-indentation depth for one of the points under the Indenter test.

The partial output values related to each specimen in Table 10 are presented in Appendix A (see Fig. 22). Fig. 23 shows the diagram of the force-indentation depth for one of the points under the Indenter test.

### 3.3 Results of theoretical analysis

Using relation (6) for the ratio of the elastic limit moment, we will have:

(18) $$M_{e}=68.58\;N.m$$

Assuming that:

(19) $$M_{1}=100\;N.m$$

Therefore, the thickness introduced into the plastic phase will be obtained using the relation (20):

(20) $$\frac{h_{1}}{c}=0.6638$$

(21) $$\Rightarrow h_{1}=0.003319$$

By substituting the abovementioned data in the obtained relations, the initial residual stress in the beam is obtained as follows:

(22) $$\sigma_{\text{ir}}=1.92\times 10^{11}\times y-9.44\times 10^{10}\times y,0\leq y\leq 0.0017$$

(23) $$\sigma_{\text{ir}}=3.21\times 10^{8}-9.26\times 10^{10}\times y,0.0017\leq y\leq 0.005$$

The thickness of the plastic when the reverse load ($h_{2}$) is applied will be obtained using relation (18). If:

(24) $$M_{2}=65\;N.m$$

*For* $h_{2}$ *we will have:*

(25) $$\frac{h_{2}}{c}=0.228$$

(26) $$\Rightarrow h_{2}=0.0011$$

Therefore, according to relations (24) and (26), the final residual stress after eliminating the moment $M_{2}$ will be obtained as follows:

(27) $$\sigma_{fr}=8.63\times 10^{10}\times y,0\leq y\leq 0.0017$$

(28) $$\sigma_{fr}=\left(0.99+0.051\times y\right)\times 324\times 10^{6}+6.14\times 10^{10},0.0017\leq y\leq 0.0039$$

(29) $$\sigma_{fr}=\left(-0.99+0.735\times y\right)\times 324\times 10^{6}+6.14\times 10^{10},0.0039\leq y\leq 0.005$$

**Fig. 24:**
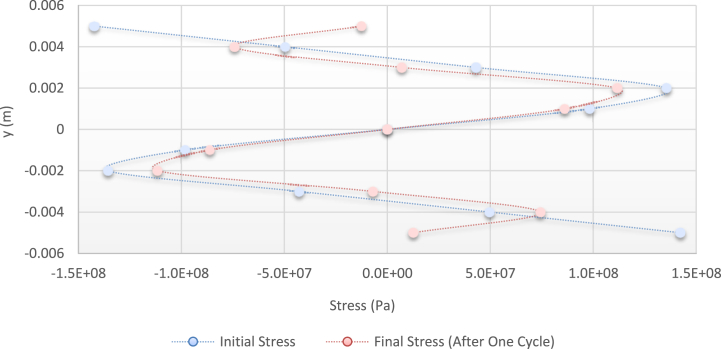
Diagram of the stress distribution in the cross-section of the beam before and after applying the alternative load (theoretical analysis).

Fig. 24 shows the stress distribution diagram obtained by the abovementioned relations.

#### 3.3.1 Comparison of results

As it is known, the results of numerical analysis and simulation are similar to each other in terms of reduction of residual stresses and their difference is only in terms of stress values. Various reasons can be mentioned to justify this difference, including the solution method used in the finite element environment, boundary conditions, and load application method. In the simulation section, the three main parameters of time, frequency, and amount of torque have been extensively tested. These results have been analysed in terms of different parameters below.

- **Frequency**: According to the results obtained in the simulation section, it can be concluded that frequency is one of the determining factors in reducing the residual stress; In such a way that if its optimal value is found for a sample, increasing the frequency further will not only have a positive effect on the work process, but considering other conditions, it can act as a relatively destructive factor against the stress relief process.
- **Torque**: As can be seen from the results, for both sexes, increasing the torque has improved the results. It is true that there is an optimal limit for this parameter, but what is more noticeable is that if the other parameters are chosen correctly, increasing the torque will have better results in the process of reducing the residual stress.
- **Time**: According to the materials mentioned in the previous chapters, most of the stress relief is done in the initial cycles, which has shown itself again in the finite element analysis results. The point that should be mentioned here is that in longer times, for most experimental situations, the results tend to lower and even negative values.

In fact, with increasing time and increasing the number of cycles, not only complete stress relief has not been achieved, but the results have shown the opposite behavior of this. To justify this article, it should be pointed out that the positive effect of increasing time can be accepted when other parameters are within the normal range or at least close to their optimal value; Otherwise, the time factor cannot be analysed and its effect cannot be studied.

What is clear and specific for the time parameter is that the initial cycles always have more effect than the later cycles, and this has been proven in the simulation phase as well.

## 4 Conclusion

The conducted research is an effort towards a deeper understanding of reducing the residual stresses of members subjected to alternating loads or vibrations in general. In the theoretical part, the theorem of the mathematical model related to plasticity and kinematic hardness is used. Also, in the simulation section, the finite element method is used to describe the material's behavior and the model's dynamic behavior. Also, octagonal, isoparametric, and explicit solution elements have been used for finite element analysis. In the experimental part, considering the same conditions as the theory and simulation part, after rolling and creating residual stress, the target workpiece is subjected to vibration load by the target mechanism, and the mechanical parameters are extracted.

As seen, the theoretical analysis results have an acceptable agreement with the simulation and experimental results.

This research yields the following results.

- When the geometry of an object is known and the stress distribution is partially determined, the mechanical vibrations almost eliminate the residual stresses on the metal surface.
- The amount of residual stress reduction can be predicted with material stress-strain properties by using simulation and analysis.
- The results of the numerical analysis and simulation have good similarities with each other in terms of residual stresses, and they differ from each other only in terms of stress values. Different reasons can be proposed to justify this difference among them are the method used in the simulation environment, boundary conditions, and load application method.
- Frequency is one of the determining factors in reducing residual stress. If the optimal value of the frequency is found for a specimen, a further increase in the frequency not only does not have a positive effect on the process but also adversely affects the stress relief process.
- Although there is an optimal level for the moment (torque) parameter, what is more, noticeable is that an increase in the moment will yield better results in the process of residual stress reduction if other parameters are well selected.
- As a general conclusion, this research expresses the basic points of the residual stress reduction mechanism when alternative loading is applied, and this mechanism can be used as a fundamental step toward more extensive research to obtain clearer insight.

It is suggested that more extensive research be done on the mechanism of reducing the residual stress of materials subjected to alternating loads. Here are some of the suggestions that can be made in this case.

- In this project, a model based on plasticity with linear kinematic stiffness was made to explain the phenomenon of vibrational stress relief. Future works can include the concept of combined kinematic and isotropic hardness, in which displacement and expansion of the initial tolerance level are considered. Also, other physical aspects of the material, such as the dependence of its behavior on the strain rate, can be considered.

The items related to the analysis model can be generalized to other materials that have different tensile and compressive yield stress.

- It is clear that the stress distribution used in this work is different from the stress distribution after the casting or rolling processes. Therefore, to create the primary residual stress that is created through these processes and is symmetrical with respect to the neutral subject, Finite element analysis softwares are used, the effect of intermittent loading is observed on them. May put rotating or different geometries from the square in the cross section of the analysis.
- It is suggested to consider different mechanisms, in addition to the vibrator, to apply vibration to the work piece so that the effect of vibration and its centers can be analysed through cheaper and more practical equipment on the parts made in different ways. These methods can also include methods of applying ultrasonic vibrations.
- It is suggested to investigate and analyze the combined effect of vibration and thermal processes in relation to each of the processes independently.

## Ethical Approval

This paper does not contain any studies with human participants or animals performed by any of the authors.

## Funding statement

The authors received no financial support for the research and/or authorship of this article.

## Availability of data and material

The data that support the findings of this study are available from the corresponding author upon reasonable request.

## CRediT authorship contribution statement

**Hamidreza Mohammadhoseini Servak:** Methodology, Investigation, Funding acquisition, Formal analysis, Data curation, Conceptualization. **Mehdi Jafari Vardanjani:** Writing – original draft, Visualization, Validation, Supervision, Software, Resources, Project administration, Methodology, Investigation, Funding acquisition, Formal analysis, Data curation, Conceptualization. **Shahrouz Yousefzadeh:** Methodology, Investigation, Funding acquisition, Formal analysis, Data curation, Conceptualization.

## Declaration of competing interest

The authors declare that they have no known competing financial interests or personal relationships that could have appeared to influence the work reported in this paper.

If there are other authors, they declare that they have no known competing financial interests or personal relationships that could have appeared to influence the work reported in this paper.

## Figures and Tables

**Table A1:** The results of the 5 tested points for the raw sample

Point number	Hardness (Vickers)	Elasticity coefficient (GPa)
1	297.636	98.191
2	166.997	77.973
3	179.376	82.049
4	253.876	99.537
5	181.073	79.234

**Table A2:** The results of the 5 tested points for the rolled sample

Point number	Hardness (Vickers)	Elasticity coefficient (GPa)
1	408.46	131.72
2	230.785	92.201
3	166.666	76.868
4	199.548	83.267
5	148.471	76.951

**Table A3:** The results of the 5 tested points for the stress relief sample

Point number	Hardness (Vickers)	Elasticity coefficient (GPa)
1	172.678	83.376
2	158.291	79.25
3	164.129	79.472
4	140.201	71.852
5	157.611	82.362

## Appendix A

The results obtained from the nano penetration test.

The results of the hardness test for a set using the Nano indenter device are presented inTables A1, A2 and A3.
